# Is the measurement of ethmoid sinus dominance in eosinophilic chronic rhinosinusitis accurate?

**DOI:** 10.1016/j.bjorl.2024.101463

**Published:** 2024-07-09

**Authors:** Kosuke Akiyama, Yasushi Samukawa, Hiroshi Hoshikawa

**Affiliations:** Kagawa University, Faculty of Medicine, Department of Otolaryngology, Kita-gun, Miki-cho, Japan

**Keywords:** Eosinophilic chronic rhinosinusitis, Ethmoid dominant shadow, Lund-Mackay scoring system, Zinreich scoring system, Chronic rhinosinusitis with nasal polyps

## Abstract

**Objective:**

An ethmoid-dominant shadow on computed tomography is an indicator of type 2 inflammation, and is one of the main items used to diagnose and classify the severity of eosinophilic chronic rhinosinusitis in the Japanese diagnostic criteria. Ethmoid sinus dominance is examined using the Lund-Mackay scoring system and may be overestimated due to scoring characteristics. We aim to investigate the accuracy of evaluations of ethmoid dominance using the conventional scoring system and the possibility of conducting an objective evaluation using a more detailed other scoring system.

**Methods:**

Patients diagnosed with eosinophilic chronic rhinosinusitis and who underwent bilateral endoscopic sinus surgery were enrolled in the present study. Computed tomography was performed preoperatively on all subjects. The bilateral anterior and posterior ethmoid sinuses and bilateral maxillary sinus were scored, and the ethmoid-to-maxillary ratio was calculated using 3 different scoring systems: Lund-Mackay (each sinus score ranges between 0 and 2), simplified Zinreich (score ranging between 0 and 3), and Zinreich (score ranging between 0 and 5).

**Results:**

A total of 149 patients were eligible for the present study. Significant differences were observed in ethmoid-to-maxillary ratio evaluated by the 3 different scoring systems (2.4 ± 0.7, 3.0 ± 1.1, and 3.7 ± 2.2). Only 2 patients were negative for ethmoid dominance by the Lund-Mackay scoring system, while 14 were negative by the simplified-Zinreich and Zinreich scoring systems. Severity changed from the initial grade in 12 patients.

**Conclusions:**

The present results confirmed a potential overestimation when only the Lund-Mackay scoring system was used to assess ethmoid dominance. Ethmoid dominance has been identified as one of the main predictive factors for the long-term postoperative outcomes of eosinophilic chronic rhinosinusitis and is included in the Japanese diagnostic criteria. A detailed evaluation of ethmoid dominance is desirable for more accurate evaluations of the severity and prognosis of eosinophilic chronic rhinosinusitis.

**Level of evidence:**

4.

## Highlights

• Ethmoid-dominant shadow on computed tomography is an indicator of type 2 inflammation.
• Ethmoid-dominance is usually evaluated by Lund-Macay scoring system.
• Its accuracy has been still unclear comparing with more detailed scoring systems.
• Potential overestimation is indicated when evaluation is done by only the Lund-Macay.
• Ethmoid dominance should be assessed using more detailed scoring systems.

## Introduction

Eosinophilic Chronic Rhinosinusitis (ECRS) is a type 2 dominant disease, a subtype of CRS with nasal polyps, and may sometimes be refractory to treatment.^1^ In Japan, an extensive multicenter survey called “The Japanese Epidemiological Survey of Refractory Eosinophilic Chronic Rhinosinusitis (JESREC) study” was conducted in 2015.^2^ The findings obtained showed that bilateral disease, the presence of nasal polyps, and an ethmoid-dominant shadow on CT were factors associated with refractoriness, and clinical diagnostic criteria for ECRS were developed based on these factors and are now commonly used in Japan. The presence of ethmoid-dominant inflammation was one of the main measures used in evaluations along with bilateralness, the presence of nasal polyps, or the percentage of blood eosinophils. Among these criteria, ethmoid dominance was assessed as the Ethmoid-to-Maxillary ratio (E/M) on preoperative Computed Tomography (CT) using the Lund-Mackay (L-M) scoring system, and was positive at E/M ≥ 2.^2^ Although the L-M scoring system is the most common and simple evaluation method, the range of 1 point is very wide and, hence, a slight shadow or air inclusion will be scored as the same point.^3^ Therefore, a scale with E/M ≥ 2 may not fully reflect ethmoid sinus dominance, and it is sometimes difficult to make a decision. The present study examined the accuracy of evaluations of ethmoid dominance using the L-M scoring system and investigated the possibility of conducting an objective evaluation using a more detailed other scoring system.

## Methods

### Study population

This retrospective study was conducted between May 2014 and December 2022. One hundred and seventy-nine consecutive patients diagnosed with ECRS and who underwent bilateral ESS at Kagawa Medical University were enrolled. All patients were scored according to the JESREC scoring system and nasal polyp biopsy was performed prior to or during surgery. A definitive diagnosis of ECRS was reached according to the JESREC diagnostic criteria based on a JESREC score ≥11 and ≥70 eosinophils per high-power field in an average of three eosinophil-rich regions. Severity was also defined according to the JESREC severity algorithm (Table 1).^1^ The baseline characteristics of patients were obtained from their medical records, including blood sampling tests, a history of allergic rhinitis, bronchial asthma, Non-steroidal anti-inflammatory drug-Exacerbated Respiratory Disease (NERD), or eosinophilic otitis media. Exclusion criteria were as follows: age <20 years and patients with a previous history of sinus surgery or receiving continuous systemic steroids or biologics at the time of surgery. This study design was approved by the Institutional Review Board, Kagawa-Medical University (approval nº 2023-068).

**Table 1:** JESREC score criteria and severity algorithm for the diagnosis of ECRS and its severity.

Factors		Score
Disease side: both sides		3
Presence of nasal polyps		2
CT shadow: ethmoid ≥ maxillary		2
Peripheral eosinophil, %		
>2% ≤5%		4
>5% ≤10%		8
>10%		10
**A**	① >5% eosinophils in peripheral blood	
	② Ethmoid-dominant shadow on CT	
**B**	① Comorbidity of bronchial asthma	
	② Aspirin intolerance	
	③ NSAIDs intolerance	
**Mild**	At least one factor A is not applied + all three factors of B are not applied	
**Moderate**	All two factors of A are applied + all three factors of B are not applied	
**Moderate**	At least one factor of A is not applied + at least one factor of B is applied	
**Severe**	All two factors of A are applied + at least one factor of B is applied	
(Complicated cases of eosinophilic otitis media are judged as severe)		

The upper table indicates ECRS diagnostic items and points. More than or equal to 11 points corresponds to ECRS. The lower table indicates the severity algorithm. JESREC, The Japanese Epidemiological Survey of Refractory Eosinophilic Chronic Rhinosinusitis; ECRS, Eosinophilic Chronic Rhinosinusitis; NSAID, Non-Steroidal Anti-Inflammatory Drug.

### Calculation of E/M

CT was performed preoperatively on all subjects. Their bilateral anterior and posterior ethmoid sinuses and bilateral maxillary sinus were scored using the following systems: the Lund-Mackay CT scoring system (each sinus score ranges between 0 and 2^3^ and the Zinreich scoring system (each sinus score ranges between 0 and 5).^4^ We also conducted measurements using the original scoring system, namely, the simplified Zinreich (S-Zinreich) scoring system: 0 = 0%, 1 = 1%–50%, 2 = 51%–99%, and 3 = 100%. Scores in each evaluation method are shown in Table 2. Scoring was performed by 2 experienced surgeons (K.A and Y. S) using thin-section sinus CT images in both the axial and coronal planes. E/M was calculated as follows: (right anterior and posterior ethmoid sinus score + left anterior and posterior ethmoid sinus score)/(right maxillary sinus score + left maxillary sinus score). Correlation coefficients between the L-M score and the S-Zinreich or Zinreich score were evaluated by Spearman’s rank test.

**Table 2:** Contents of each scoring system.

	Score and shadow occupancy rate
L-M	0 = 0%, 1 = 1 %–99%, 2 = 100%
S-Zinreich	0 = 0%, 1 = 1 %–50%, 2 = 51%–99%, 3 = 100%
Zinreich	0 = 0%, 1 = 1%–25%, 2 = 26 %–50%, 3 = 51%–75%
	4 = 76%–99%, 5 = 100%

L-M, Lund and Mackay scoring system; S, Simplified.

### Assessment of the presence/absence of ethmoid dominance

The presence or absence of ethmoid dominance was evaluated stepwise using the 3 scoring systems. E/M > 2 or < 2 by the L-M scoring system was confirmed. In cases with E/M = 2, images were re-evaluated by the S-Zinreich scoring system. Ethmoid sinus shadow was dominant when the ethmoid and maxillary sinuses were both completely filled bilaterally and E/M = 8/4 = 2. Patients were divided into 3 patterns. The E/M = 2 group by the S-Zinreich scoring system was subjected to further assessments using the Zinreich scoring system. The remaining patients were divided into the E/M ≥ 2 or E/M < 2 group by the Zinreich scoring system and were evaluated as positive or negative for ethmoid dominance.

### Postoperative course evaluation

The short-term administration of Oral Corticosteroids (OCS), a poor prognosis, and a history of secondary treatment, including revision surgery and biologics for ECRS, were examined as postoperative long-term outcomes. Patients with a history of prescriptions even once for exacerbation in the upper or lower airways during the postoperative follow-up, except for the perioperative period, were considered to use OCS. Patients with a poor mucosal condition, those in whom it was difficult to observe the middle meatus without improvement, and those with persistent strong subjective symptoms, such as olfactory disturbance and nasal congestion, at the time of the final observation failed to achieve disease control (poor prognosis).

### Statistical analysis

Statistical analyses were performed with EZR (Saitama Medical Center, Jichi Medical University, Saitama, Japan). Comparisons of results between each group were performed using the Student’s *t*-test or Fisher’s exact test. Correlation coefficients between the L-M scoring system and other scoring systems were evaluated by Spearman’s rank test. The significance of differences was assumed when *p* < 0.05. In multiple comparisons of 3 or more groups, the significance level was defined as *p* < 0.05/number of groups.

## Results

### Patient characteristics and CT scoring

Twenty-eight patients with a previous history of sinus surgery and 2 who were receiving continuous OCS or biologics at the time of surgery were excluded from the assessment. A total of 149 patients were eligible for the present study. Baseline characteristics are shown in Table 3. The severity of ECRS was mild in 12 patients (8%), moderate in 55 (37%), and severe in 82 (55%).

**Table 3:** Baseline characteristics of patients.

	n = 149
Age (years, mean ± SD)	52.5 ± 11.9
Sex (male, female)	74, 75
Allergic rhinitis	72 (48.3%)
Bronchial asthma	100 (67.1%)
NERD	11 (7.4%)
Eosinophilic otitis media	5 (3.4%)
CT score (mean ± SD)	15.2 ± 5.0
Total IgE (IU/mL, mean ± SD)	413 ± 762
White blood cells (10^3^/μL, mean ± SD)	6360 ± 1708
Percentage of blood eosinophils (%)	8.6 ± 4.2
JESREC score (11, 13, 15, and 17)	31, 6, 77, 35
Severity (mild, moderate, and severe)	12, 55, 82

NERD, Non-steroidal anti-inflammatory drug-Exacerbated Respiratory Disease; JESREC, The Japanese Epidemiological Survey of Refractory Eosinophilic Chronic Rhinosinusitis.

CT scores by the L-M, S-Zinreich, and Zinreich scoring systems were 5.2 ± 1.5, 7.9 ± 2.6, and 13.4 ± 5.6, respectively, in the ethmoid sinus and 2.2 ± 0.6, 2.9 ± 1.3, and 4.5 ± 2.5, respectively, in the maxillary sinus. E/M were 2.4 ± 0.7, 3.0 ± 1.1, and 3.7 ± 2.2, respectively, with significant differences between each of the groups (Table 4A). A strong correlation was observed between L-M scores and other scores in the ethmoid sinus and maxillary sinus (*r* = 0.758 to 0.873). Significant values were also observed in E/M with a moderate correlation between L-M and other scoring systems (Table 4B).

**Table 4:** CT scores in each scoring system and correlation coefficients.

A			
	Ethmoid / Maxillary sinus	E/M	95%CI
L-M	5.2 ± 1.5 / 2.2 ± 0.6	2.4 ± 0.7	2.287‒2.513
S-Zinreich	7.9 ± 2.6 / 2.9 ± 1.3	3.0 ± 1.1^a^**	2.822‒3.178
Zinreich	13.4 ± 5.1 / 4.5 ± 2.5	3.7 ± 2.2^b^**,^c^**	3.344‒4.056

B		
	Correlation coefficient (95% CI)	*p*-value
**Ethmoid sinus**		
S-Zinreich	0.873 (0.829‒0.907)	*p* < 0.001**
Zinreich	0.791 (0.722‒0.845)	*p* < 0.001**
**Maxillary sinus**		
S-Zinreich	0.826 (0.767‒0.871)	*p* < 0.001**
Zinreich	0.758 (0.681‒0.819)	*p* < 0.001**
**E/M**		
S-Zinreich	0.525 (0.398‒0.632)	*p* < 0.001**
Zinreich	0.473 (0.338‒0.589)	*p* < 0.001**

(A) CT scores and ethmoid-to-maxillary sinus ratios evaluated by each scoring system. (a, b, c) Mean p-values between L-M and S-Zinreich, L-M and Zinreich, and S-Zinreich and Zinreich.

(B) Correlation coefficients between L-M and the S-Zinreich and Zinreich scoring systems. L-M, Lund and Mackay scoring system; S, Simplified; E/M, Ethmoid-to-Maxillary sinus ratio; CI, Confidence Interval.

** *p* < 0.01.

### Ethmoid-dominant shadows

**Fig. 1:**
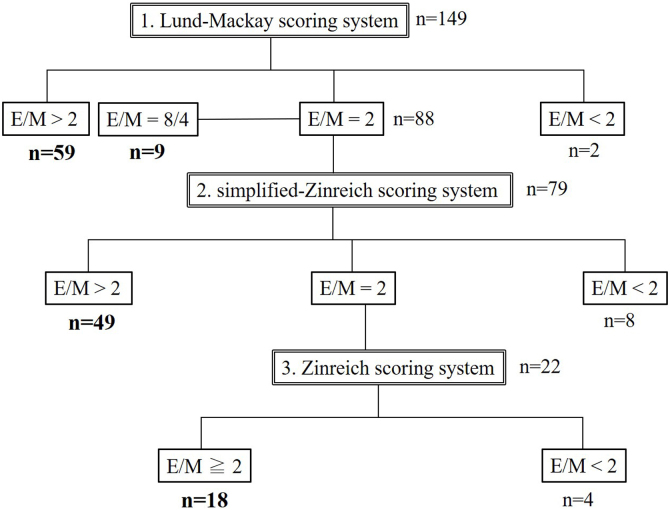
Chart of an assessment of ethmoid sinus dominance. E/M, Ethmoid-to-Maxillary sinus ratio.

**Fig. 2:**
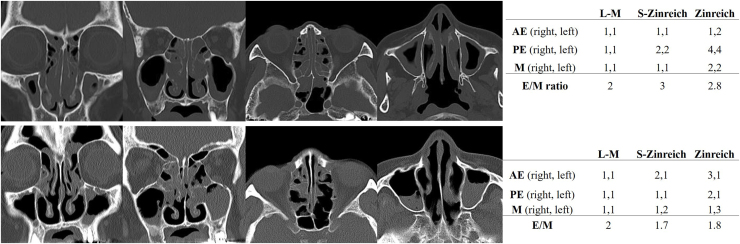
CT images showing different evaluation patterns. Axial and coronal bone window CT images. The upper case shows the presence of ethmoid-dominant shadow. The lower case shows the absence of ethmoid-dominant shadow. AE, Anterior Ethmoid Sinus; PE, Posterior Ethmoid Sinus; M, Maxillary Sinus; E/M, Ethmoid-to-Maxillary sinus ratio; L-M, Lund and Mackay scoring system; S, Simplified.

Judgements on ethmoid dominance were performed stepwise using the 3 scoring systems. The initial evaluation (L-M scoring system) identified 147 (E/M ≥ 2) and 2 (E/M < 2) subjects. After excluded 9 subjects whose E/M = 8/4 = 2, 79 subjects with E/M = 2 were re-evaluated by the S-Zinreich scoring system, and 49 positive (E/M > 2) and 8 negative subjects (E/M < 2) were identified. The remaining 22 subjects (E/M = 2) were divided into 18 positive (E/M ≥ 2) and 4 negative (E/M < 2) subjects by the Zinreich scoring system (Fig. 1). Overall, 135 subjects were identified as positive and 14 as negative for ethmoid dominance. Representative cases are shown in Fig. 2. The severity grading of ECRS was revised based on the results obtained, and it changed in 12 patients from the initial grade; 4 changed from severe to moderate, 4 from moderate to mild, 2 from moderate to non-ECRS, and 2 from mild to non-ECRS. Initial and revised severity percentages are shown in Table 5.

**Table 5:** ECRS severity.

	L-M	Zinreich
Non-ECRS	0	4 (2.7)
Mild	12 (8.1%)	14 (9.4%)
Moderate	55 (36.9%)	53 (35.6%)
Severe	82 (55.0%)	78 (52.3%)

ECRS severity before and after an assessment of ethmoid sinus dominance. ECRS, Eosinophilic Chronic Rhinosinusitis.

### Evaluation of postoperative outcome

Postoperative long-term outcomes were evaluated by initial and revised severities (Table 6A). The average observation period was 34.3 ± 29.6 months (3–105 months). The overall numbers of subjects using OCS, with a poor prognosis, and a history of secondary treatment were 55 (36.9%), 27 (18.1%), and 22 (14.8%), respectively. The highest rates for all 3 items were observed in the severe ECRS group. In contrast, only one patient required OCS and no patients had a poor prognosis or required secondary treatment in the mild group. No significant changes were observed in comparisons of the initial and revised results in each severity group. Additionally, only 1 patient used OCS, and none had a poor prognosis or secondary treatment among 14 patients identified as negative for ethmoid dominance (Table 6B).

**Table 6:** Long-term outcomes before and after assessments of E/M.

A						
	OCS use		Poor prognosis		Secondary treatments	
	L-M	Zinreich	L-M	Zinreich	L-M	Zinreich
Mild (or non-ECRS)	0/12 (0%)	1/18 (5.6%)	0/12 (0%)	0/18 (0%)	0/12 (0%)	0/18 (0%)
Moderate	11/55 (20.0%)	11/53 (20.8%)	4/55 (7.3%)	4/53 (7.6%)	2/55 (3.6%)	3/53 (5.7%)
Severe	44/82 (53.7%)	43/78 (55.1%)	23/82 (28.0%)	23/78 (29.5%)	20/82 (24.4%)	19/78 (24.4%)
Overall	55/149 (36.9%)		27/149 (18.1%)		22/149 (14.8%)	

B			
	OCS use	Poor prognosis	Secondary treatments
Ethmoid dominance			
Absence (n = 14)	1 (7.1%)	0	0
Presence (n = 135)	54 (40.0%)	27 (20.0%)	22 (16.3%)
p-value	0.018^a^	0.075	0.249

(A) Long-term outcomes by severity as assessed by the Lund and Mackay or Zinreich scoring system.

(B) Comparison of long-term outcomes with and without ethmoid dominance by the Zinreich scoring system. Secondary treatments indicate the requirement for revision surgery or biologics.

E/M, Ethmoid-to-Maxillary sinus ratio; OCS, Oral Corticosteroid; ECRS, Eosinophilic Chronic Rhinosinusitis.

a *p* <  0.05.

## Discussion

Evidence to show that an ethmoid-dominant shadow on CT is an indicator of type 2 inflammation was initially obtained in Japan.^5^ Similar studies were subsequently reported in succession from other Asian countries, and their findings suggested that the postoperative course was more likely to be poor in patients with high E/M.^6^, ^7^, ^8^ Equivalent findings were obtained in the JESREC study, in which an ethmoid-dominant shadow was adopted as one of the main diagnostic factors because it was an independent predictor of refractoriness and postoperative recurrence.^1^, ^2^ Therefore, the assessment of ethmoid dominance is mandatory to reach a definitive diagnosis of ECRS and is also indispensable for severity classifications when the JESREC criteria are used for a diagnosis.

The L-M scoring system is commonly used in CT evaluations of CRS. Although the L-M scoring system is very simple and easy to understand, it may not sufficiently reflect the degree of inflammation due to its wide range of 1 point and the reliability of whether E/M = 2 accurately reflects ethmoid dominance in all cases. Therefore, we examined E/M in 3 steps using different scoring systems, and investigated the accuracy of an assessment of ethmoid sinus dominance using the L-M scoring system (Fig. 1). The Zinreich scoring system was devised as a modification of the L-M scoring system and has 6 graded subdivisions.^4^ However, the use of a detailed grading system has the disadvantage of being complicated and ratings may vary among graders. Therefore, we added a 4-grade scoring system that simplified the Zinreich scoring system in the present study. In comparisons of these scoring systems with the L-M scoring system, the scores in each sinus strongly correlated, which is consistent with previous findings.^9^ On the other hand, although a correlation was observed in E/M, averages were 2.4 ± 0.7, 3.0 ± 1.1, and 3.7 ± 2.2, showing significant differences among the 3 scoring systems. Among the 79 cases with E/M = 2 by the L-M scoring system, 8 cases were defined as ethmoid dominant negative by the S-Zinreich scoring system. Further detailed analyses using the Zinreich system revealed that additional 4 cases were negative for ethmoid dominance. Only 2 cases initially showed no ethmoid sinus dominance, and an overestimation was suggested when only the L-M scoring system was used. Twelve out of 79 cases (15.2%) were found to be false positives by a detailed evaluation, indicating errors with the L-M method due to the scoring system itself. On the other hand, the possibility of a subjective bias in detailed evaluation methods cannot be ruled out. Okushi et al. reported that the Zinreich method produced a difference in scores of ±1 in approximately 25% of cases when subjective scores were compared with digitally analyzed soft tissue density areas.^10^ However, only a few cases showed an obvious difference in scores of ±2 or more, and they concluded the more efficient ability of a modified scoring system to grade cases with clinically acceptable accuracy. The ability of the S-Zinreich scoring system used in the present study to evaluate cases was similar to that of the Zinreich system. In addition, this method mainly shows whether the soft tissue density rate is greater than or less than 50%, and is considered to be even less likely to have a subjective bias than the Zinreich scoring system. Although the L-M method almost never had a subjective bias, errors caused by the system are inevitable; therefore, the S-Zinreich method more accurately assesses the actual state of inflammation with a relatively small subjective error. Based on these findings, one of these methods may be used for a reliable assessment of ethmoid dominance when it is difficult to judge using the L-M scoring system.

When the severity of ECRS was re-evaluated after precise assessments, the severity grade was downgraded in 12 cases, including 4 cases that did not meet the diagnostic criteria for ECRS. The JESREC criteria are defined not only by ethmoid sinus dominance, but also by the combination of other recurring factors, including the peripheral blood eosinophil ratio and comorbidity of bronchial asthma, and they are considered to accurately reflect the long-term prognosis of patients.^2^, ^11^ We investigated whether a more accurate evaluation of ethmoid sinus dominance affected results on long-term prognosis. Overall, no significant changes were observed within each severity grade for the postoperative prognostic indicators of OCS use, poor mucosal control, and the introduction of secondary treatment. The present results also showed that the positive rates of these items were markedly higher in severe cases, and, conversely, the majority of mild cases maintained a favorable long-term prognosis. In addition, the prognosis of patients with no ethmoidal sinus predominance was good and they only required minor additional treatment, such as OCS or biologics (Table 6B). Therefore, a detailed assessment of ethmoid sinus dominance may be meaningful not only in cases of ECRS diagnosed according to the JESREC criteria, but also with other criteria.

The present study has a limitation that needs to be addressed. Although scoring was performed by two experienced rhinologists, a discrepancy may have existed between the actual shadow ratio based on a digital analysis and the visual score. A digital analysis was not conducted in the present study, and the extent of any discrepancy between subjective and objective evaluations was unclear. Our case series included 2 non-typical cases (comorbid with unilateral odontogenic maxillary sinusitis and chronic non-invasive maxillary sinus mycosis) with E/M < 2 by the Zinreich scoring system. In addition, the chronic non-invasive fungal case was the only case with OCS use despite the absence of ethmoid sinus dominance. Evaluations of E/M need to be carefully considered when maxillary sinus shadow is enhanced due to these pathologies.

## Conclusion

An evaluation of ethmoid sinus dominance is essential for the correct diagnosis of ECRS using the JESREC criteria. We assessed the accuracy of ethmoid sinus dominance evaluated using the L-M scoring system by comparing it with another more detailed scoring system. The results obtained showed the potential for an overestimation when only the conventional L-M scoring system was used. The present study indicates that a detailed evaluation of ethmoid sinus dominance using methods other than the L-M scoring system is desirable for the diagnosis, severity classification, and prognostic assessment of ECRS.

## Meeting of Ethical Standards (Ethics Committee statement, if absent, justify why)

All authors comply with the Declaration of Helsinki. Our study design was approved by the Institutional Review Board, Kagawa-medical university (approval No. 2023-068).

## Conflicts of interest

The authors declare no conflicts of interest.
